# Supplementary material for: Full-spectrum nonmetallic plasmonic carriers for efficient isopropanol dehydration
Source: Nat Commun. 2022 Nov 15;13:6984. doi: 10.1038/s41467-022-34738-z (PMC9666589; doi:10.1038/s41467-022-34738-z)
Supplement: Supplementary file 1 — Supplementary Information [file 41467_2022_34738_MOESM1_ESM.pdf]

Supporting Information

Full-spectrum nonmetallic plasmonic carriers for efficient isopropanol dehydration

Changhai Lu,[1,#] Daotong You,[2,#] Juan Li,[1] Baojun Li,[1] Tuan Guo[2,3,*] and Zaizhu Lou[1*]

[1]Institute of Nanophotonics, Jinan University, Guangzhou, 511443, China

[2]Institute of Photonics Technology, Jinan University, Guangzhou, 511443, China

[3]Southern Marine Science and Engineering Guangdong Laboratory (Zhuhai), Zhuhai 519000, China.

E-mail: zzlou@jnu.edu.cn (Z. Lou); tuanguo@jnu.edu.cn (T. Guo)

Chemicals. Tungsten chloride (WCl_6, >99%) and Graphene oxide (GO, 2 mg/mL) was obtained from Sigma-Aldrich. Isopropanol (99.5%), ethanol (99.8%), propanol (99.5%) and glycerol (99.5%) were obtained from Macklin. Milli-Q water (18.2 MΩ) was used in experiment. All the chemical reagents were purchased for use without any further purification.

Synthesis of rGO and $W_{18}O_{49}$-NWs. rGO layers were synthesized from GO reduction by using sodium borohydride in solution.[1] $W_{18}O_{49}$ nanowires ($W_{18}O_{49}$-NWs) with dimeter of 10-15 nm and length of 1.5-2 μm were synthesized by using WCl_6 as precursor via solvothermal treatment.[2] TEM images of $W_{18}O_{49}$-NWs/rGO with different compositions were shown in **Fig. S1**. As the increase of rGO, WO-NWs is growth on surface of rGO in good dispersion. However, when rGO is over 1 wt%, and surface of $W_{18}O_{49}$-NWs is covered by the rGO layers which will restrict the exposure of active sites for surface catalysis.

XPS analysis. For W 4f XPS spectra (**Fig. S5**), two peaks can be divided into four bands, and in which two strong bands around 36.4 and 38.5 eV are assigned to $4f_{2/5}$ and $4f_{7/5}$ orbitals of W^{6+}, respectively.[3] Meanwhile, the other weak bands around 35.0 and 37.2 eV are assigned to W^{5+} $4f_{2/5}$ and $4f_{7/5}$, respectively.[4] The W^{5+}/W^{6+} molar ratio is calculated to 11.5% and 17.1% for plasmonic $W_{18}O_{49}$-NWs and $W_{18}O_{49}$-NWs/rGO-1%, respectively. In situ XPS spectra (**Fig. S6**) shows that W^{5+}/W^{6+} molar ratio is increased from 17.1% to 24.6% after 20 min full-spectrum light irradiation, indicating the photoelectron trapped on plasmonic $W_{18}O_{49}$-NWs, and which is consistence with the DRS spectra of Fig. 1l. During synthetic process, $W_{18}O_{49}$-NWs have preferential growth on the surface of rGO rather than self-aggregation, and which refrain from the stack of nanowires, resulting in more surface oxygen vacancies. It can be confirmed from O 1*s* XPS spectra (**Fig. S7b)**. Oxygen vacancies can absorb H_2O to generate OH

on surface.[5]

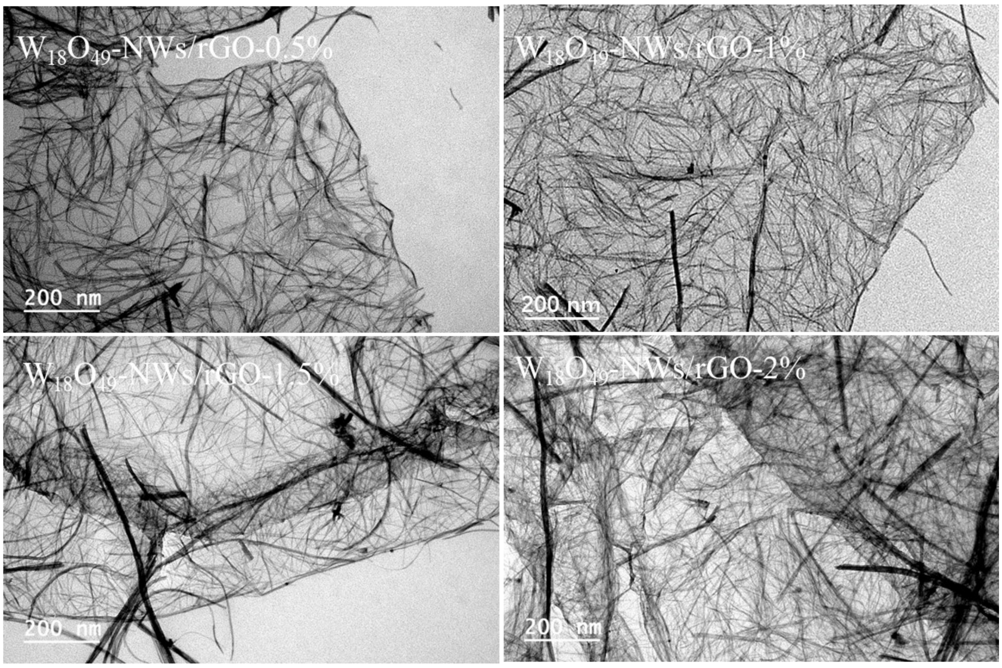

Fig. S1 TEM images of $W_{18}O_{49}$-NWs/rGO with different compositions.

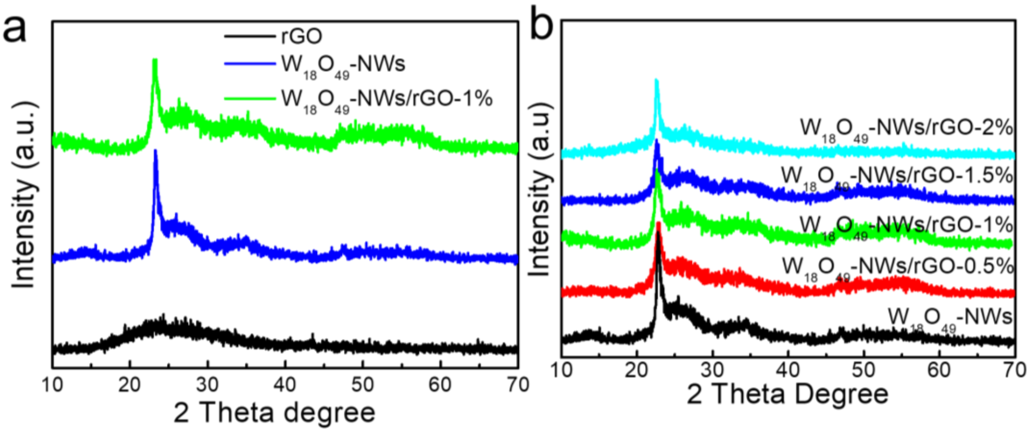

Fig. S2 XRD patterns (a) of rGO, $W_{18}O_{49}$-NWs and $W_{18}O_{49}$-NWs/rGO-1%. XRD patterns (b) of $W_{18}O_{49}$-NWs/rGO with different compositions.

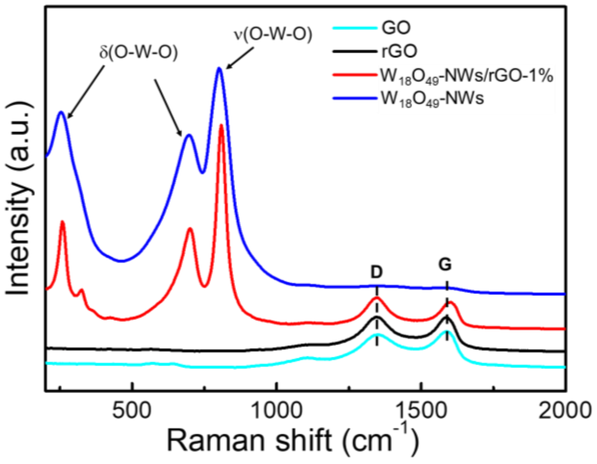

Fig. S3 Raman spectra of $W_{18}O_{49}$-NWs/rGO-1%, $W_{18}O_{49}$-NWs, rGO and GO.

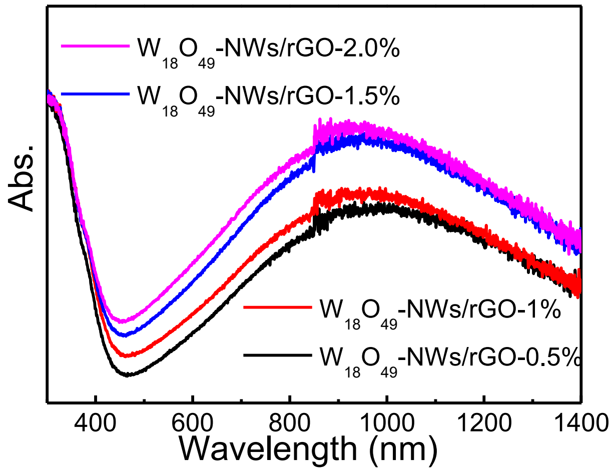

Fig. S4 UV-Vis-NIR DRS of $W_{18}O_{49}$-NWs/rGO with different compositions.

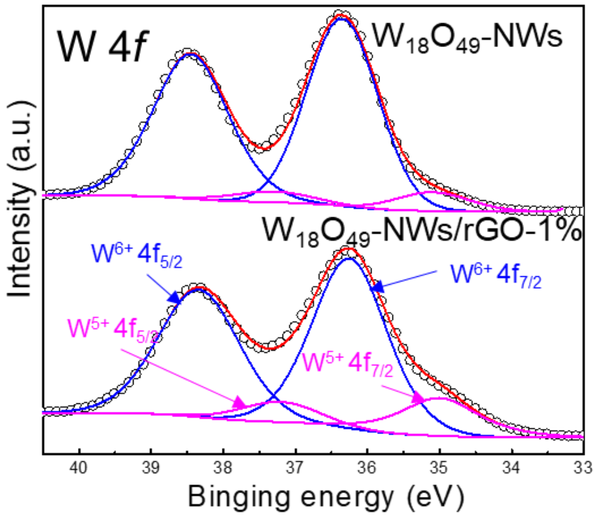

Fig. S5 W 4*f* XPS spectra of $W_{18}O_{49}$-NWs and $W_{18}O_{49}$-NWs/rGO-1%.

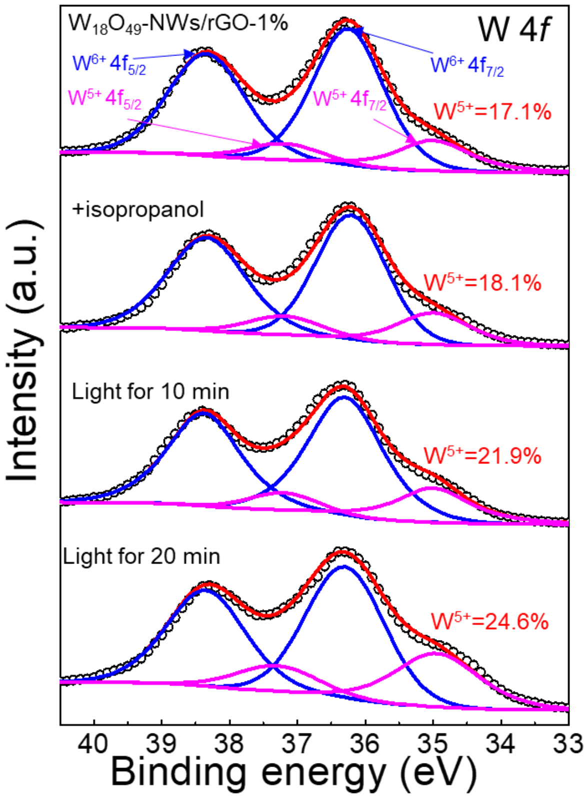

Fig. S6 In situ W 4*f* XPS spectra of $W_{18}O_{49}$-NWs/rGO-1% under full-spectrum light irradiation.

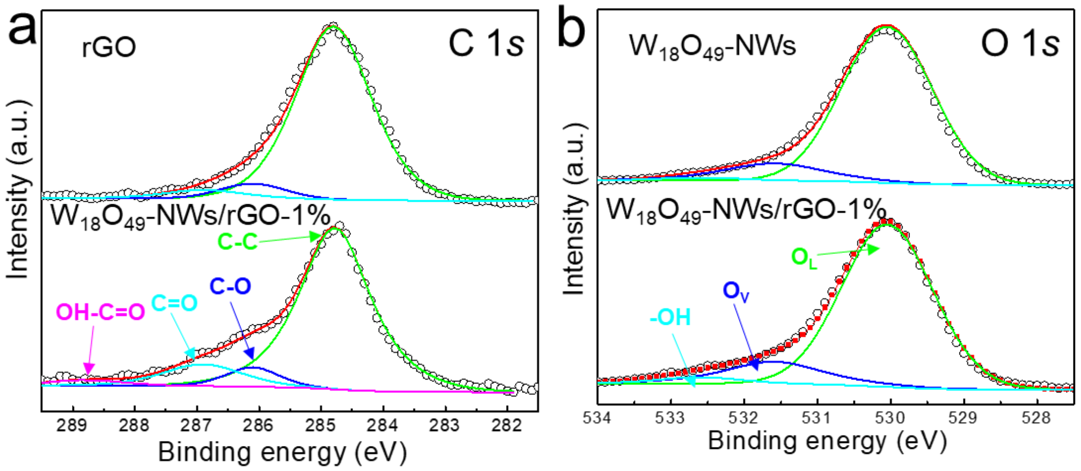

Fig. S7 XPS spectra of C 1*s* (a) and O 1*s* (b) in $W_{18}O_{49}$-NWs, rGO and $W_{18}O_{49}$-NWs/rGO-1%, respectively. O_L: Lattice oxygen. O_V: Oxygen vacancies.

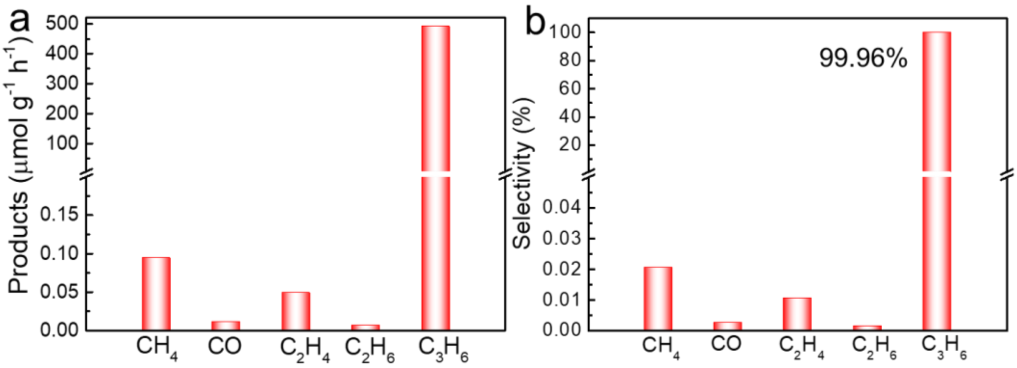

Fig. S8 Generation rates (a) and selectivity (b) of products from isopropanol dehydration reaction.

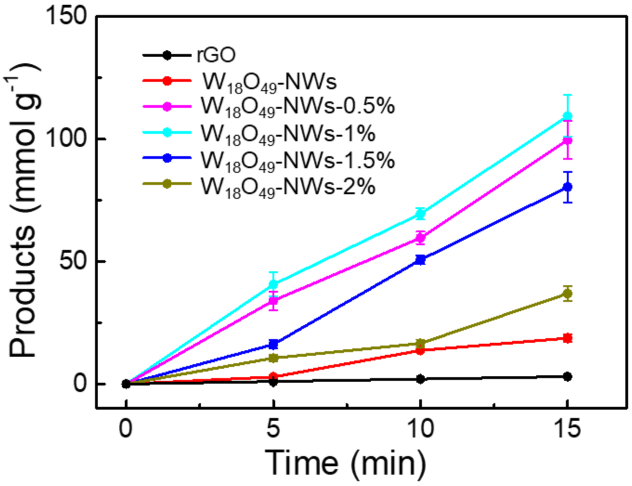

Fig. S9 Propylene generation over various $W_{18}O_{49}$-NWs/rGO heterostructure as catalysts under full-spectrum light irradiation.

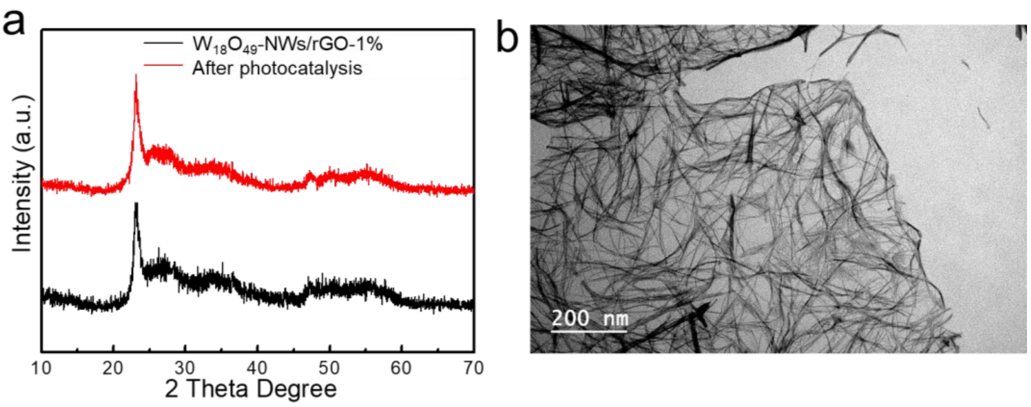

Fig. S10 XRD patterns (a) and TEM image (b) of $W_{18}O_{49}$-NWs/rGO-1% after catalysis.

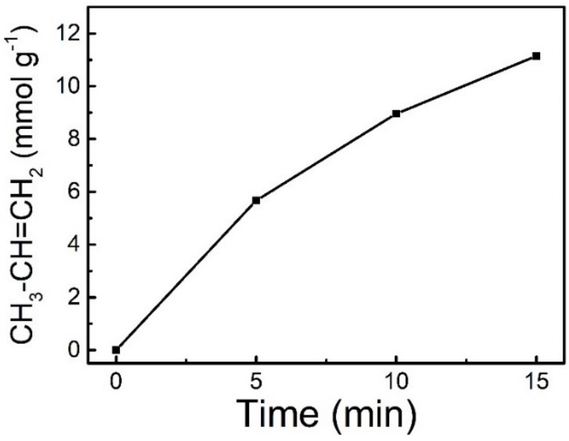

Fig. S11 Propylene generations from isopropanol/ethanol/water mixtures with a ratio of 1:1:8.

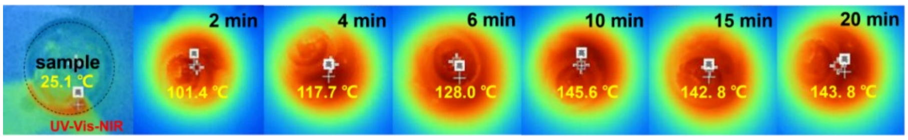

Fig. S12 UV-Vis-NIR-irradiation induces the surface temperature varies of $W_{18}O_{49}$-NWs/rGO-1%.

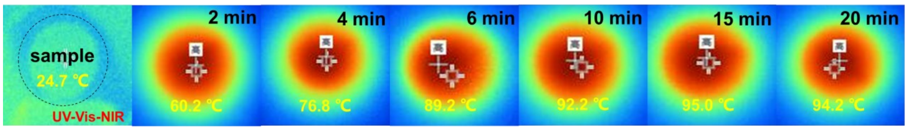

Fig. S13 UV-Vis-NIR-irradiation induces the surface temperature varies of $W_{18}O_{49}$-NWs.

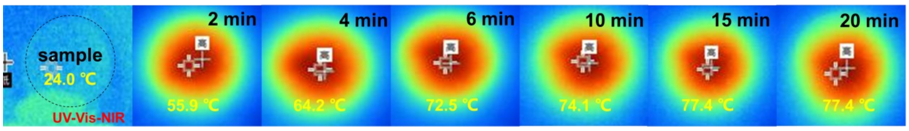

Fig. S14 UV-Vis-NIR-irradiation the induces surface temperature varies of rGO.

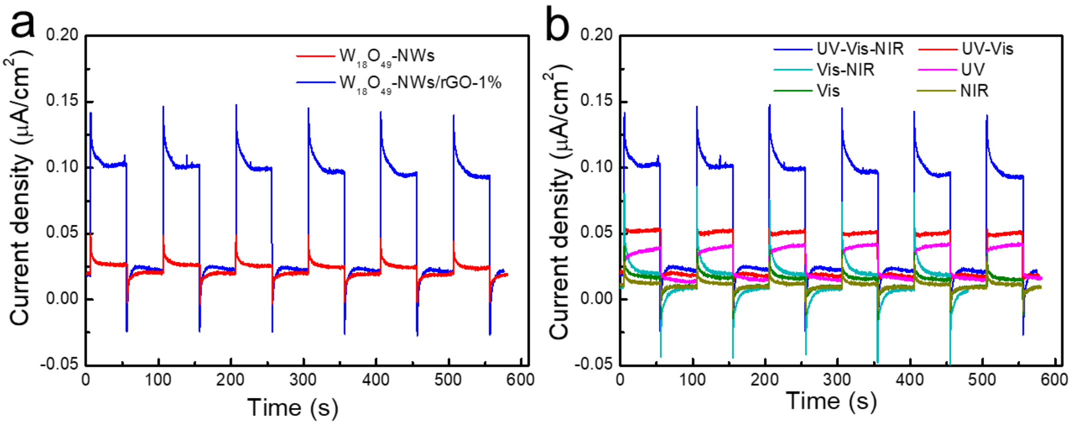

Fig. S15. a) Photocurrents of $W_{18}O_{49}$-NWs/rGO-1% and $W_{18}O_{49}$-NWs under full-spectrum light irradiation. b) Different light irradiation induced photocurrent of $W_{18}O_{49}$-NWs/rGO-1%.

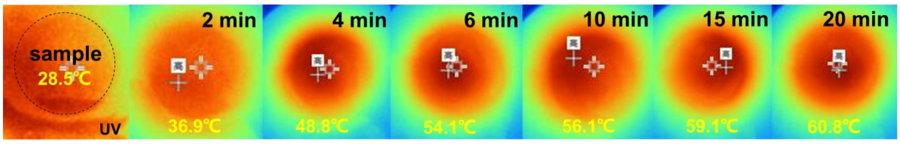

Fig. S16 UV-irradiation induces the surface temperature varies of $W_{18}O_{49}$-NWs/rGO-1%.

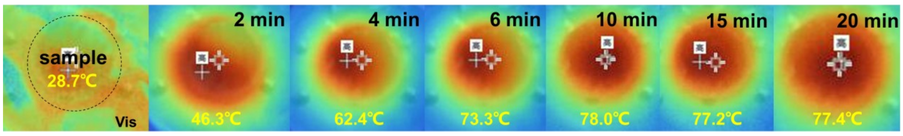

Fig. S17 Vis-irradiation induces the surface temperature varies of $W_{18}O_{49}$-NWs/rGO-1%.

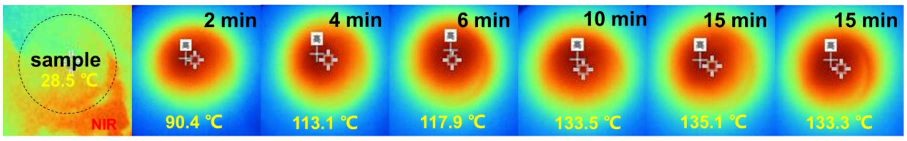

Fig. S18 NIR-irradiation induces the surface temperature varies of $W_{18}O_{49}$-NWs/rGO-1%.

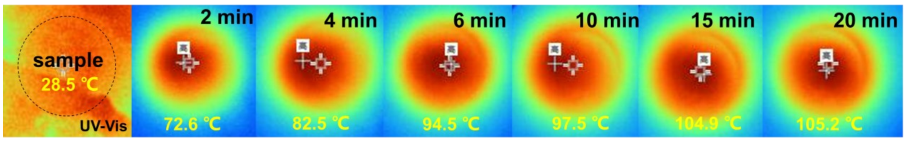

Fig. S19 UV-Vis -irradiation induces the surface temperature varies of $W_{18}O_{49}$-NWs/rGO-1%.

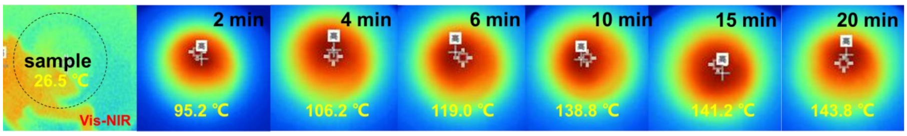

Fig. S20 Vis-NIR-irradiation induces the surface temperature varies of $W_{18}O_{49}$-NWs/rGO-1%.

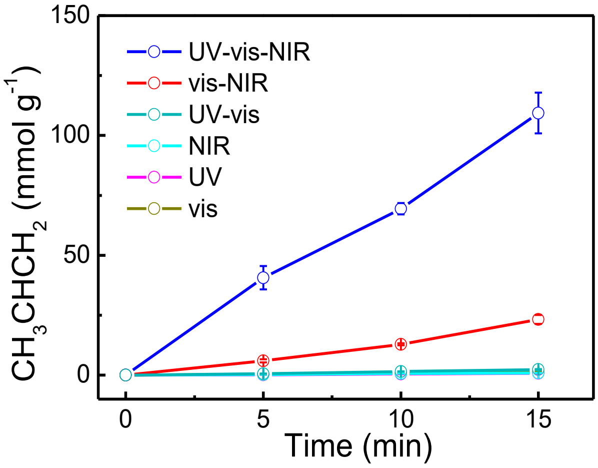

Fig. S21 Different light irradiations induce the propylene generation over $W_{18}O_{49}$-NWs/rGO-1% as photocatalyst.

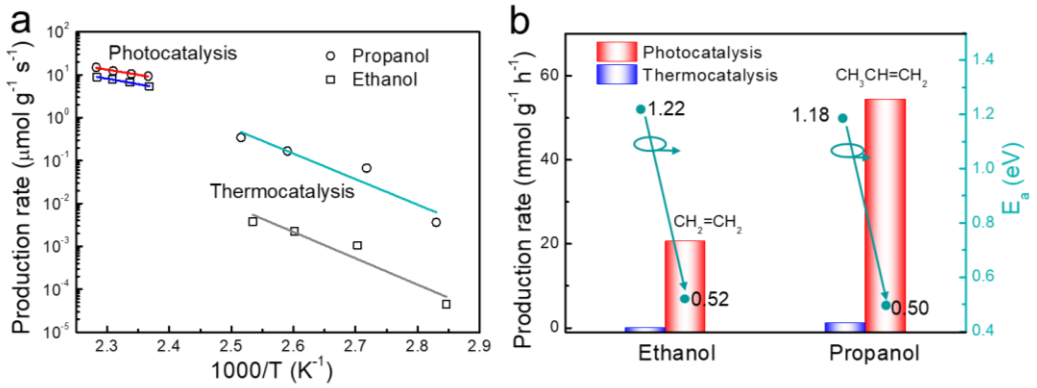

Fig. S22. a Arrhenius plots of apparent activation barriers for ethanol and propanol dehydration over $W_{18}O_{49}$-NW/rGO-1% under dark and full-spectrum light irradiation. **b** Thermocatalytic and photocatalytic ethanol and propanol dehydration rates and their calculated activation barriers (E_a).

Supplementary References

1. Guex, L. G. et al. Experimental review: chemical reduction of graphene oxide (GO) to reduced graphene oxide (rGO) by aqueous chemistry. *Nanoscale* **9**, 9562 (2017).
2. Zhang, N. et al. Refining defect states in $W_{18}O_{49}$ by Mo doping: A strategy for tuning N_2 activation towards solar-driven nitrogen fixation. *J. Am. Chem. Soc.* **140**, 9434-9443 (2018).
3. Zhang, N. et al. Defective Tungsten oxide hydrate nanosheets for boosting aerobic coupling of amines: synergistic catalysis by oxygen vacancies and brønsted acid sites. *Small 13*, 1701354 (2017).
4. Park, J. et al. Investigation of the support effect in atomically dispersed Pt on WO_{3-x} for utilization of Pt in the hydrogen evolution reaction. *Angew. Chem. Int. Ed.* **58**, 16038-16042 (2019).
5. Deng, Y. et al. Integrated full-spectrum solar energy catalysis for zero-emission ethylene production from bioethanol. *Adv. Funct. Mater.* 2110026 (2021).
